# Progressive Multifocal Leukoencephalopathy after Three Consecutive Liver Transplantations

**Published:** 2015-08-01

**Authors:** F. Ozdemir, V. Ince, A. Baskiran, Z. Ozdemir, Y. Bayindir, B. Otlu, S. Yilmaz

**Affiliations:** 1 Inonu University, Liver Transplantation Institute, Department of General Surgery, Malatya, Turkey; 2 Inonu University, Liver Transplantation Institute, Department of Radiology, Malatya, Turkey; 3 Inonu University, Liver Transplantation Institute, Department of Infectious Diseases, Malatya, Turkey; 4 Inonu University, Faculty of Medicine, Department of Medical Microbiology, Malatya, Turkey

**Keywords:** Liver transplantation, Leukoencephalopathy, progressive multifocal, JC virus, Immunocompromised host, Viral load

## Abstract

Progressive multifocal leukoencephalopathy (PML) is a lytic infection of the central nervous system caused by the reactivation of John Cunningham Virus (JCV) in severely immunosuppressed patients. Occurrence of PML after solid organ transplantations, especially after liver transplantation, is rare. If a patient has poor prognostic factors such as atypical radiological involvements or high viral load in cerebrospinal fluid (CSF), overall survival rates could be poor. Herein, we report on a patients who underwent liver transplantation three times and developed PML with unexpected radiological findings; he was also positive for JCV DNA with a high viral load. Although there are limited data about efficacy of cytarabine against JCV, it was given to the patient for five days. Despite the initiation of cytarabine and complete cessation of the immunosuppressive therapy, we lost the patient, unfortunately.

## INTRODUCTION

John Cunningham virus (JCV) is a nonenveloped icosahedral DNA virus belongs to Polyomaviridae. After primary infection, mostly occurs during childhood, the latent infection may be reactivated and cause severe demyelinating disease of the central nervous system (CNS) in immunosuppressed patients [1-3]. Progressive multifocal leukoencephalopathy (PML) is a demyelinating disease caused by the reactivation of JCV. The incidence of PML has been increased since the AIDS pandemic [5]. PML has also been described in some cases including multiple sclerosis, Crohn’s disease, rheumatoid arthritis or psoriasis as a complication of immunosuppressive therapies [1].

The manifestations of PML are usually subacute neurologic deficits including altered mental status, motor deficits, ataxia, visual symptoms, and seizure. The course of PML is usually progressive and fatal [1]. Especially in non-HIV-related PML, a 1-year survival rate can be extremely poor—about 10% [6]. Occurrence of PML after solid organ transplantation, especially after liver transplantation, is rare. Herein, we report on a patient who received liver transplant three times and developed PML with atypical radiological findings.

## CASE REPORT

A 55-year-old male patient who suffered from esophageal variceal bleeding was diagnosed with chronically liver failure due to hepatitis B. The patient underwent endoscopic band ligation 12 times on his past medical history for esophageal variceal bleeding. His Model for End-Stage Liver Disease (MELD) score was 19 at admission to our institute. The remarkable laboratory findings were total bilirubin of 5.6 mg/dL, INR of 1.4, and serum creatinine of 1.3 mg/dL. He underwent deceased-donor liver transplantation. Hepatic artery thrombosis (HAT) was occurred two days after the initial operation. Then, he underwent a second liver transplantation three days after the initial operation. Three days after the second operation, HAT was diagnosed again by doppler ultrasonography and confirmed by computed tomography (CT). We planned another emergency liver transplantation and performed it two days after the diagnosis of the second HAT. Postoperative follow-up was unremarkable. The patient was discharged with prednisolone 5 mg/day, mycophenolate mofetil 1000 mg/day, and tacrolimus 4 mg/day for immunosuppression. The blood tacrolimus level of the patient was 9 ng/mL when he was discharged. Steroid withdrawal from the immunosuppressive therapy was obtained by decreasing the daily dosage of the prednisolone six months of discharge. Patient’s follow-up was unremarkable eight months after transplantation until he was diagnosed with cholangitis due to bile duct stenosis. We planned to perform an ERCP to cannulate and clean the biliary duct. After the intervention and appropriate antimicrobial therapy, the patient got better; his elevated bilirubin levels decreased to normal.

Nine months after the last liver transplantation, the patient was readmitted to our institute with neurological symptoms such as focal weakness of his extremities and dysarthria for previous two weeks. He was partially cooperated and his orientation was poor during his first neurological examination. Muscle strength was graded at 4/5 on the right side. He had hyperactive deep tendon reflexes. Babinski and palmomental reflexes were positive, bilaterally. The patient had also mild degree of background activity disorder on electroencephalography. The liver functions were mildly elevated on laboratory tests (ALT: 136 IU/L, AST: 92 IU/L, T. Bil: 1.5 mg/dL, ALP: 250 IU/L).

**Figure 1:**
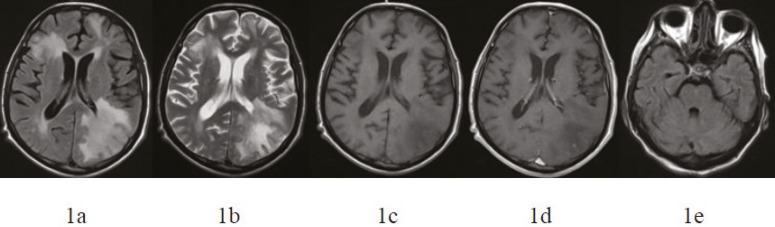
Axial fluid attenuated inversion recovery (FLAIR) (a) and axial T2-weighted images (b) show extensive hyperintense and axial T1-weighted image (c) shows hypointense lesions in the subcortical and periventricular white matter bilaterally. There is also compression of the posterior horn of the lateral ventricle due to mass effect. There is no remarkable contrast enhancement on T1-weighted postcontrast images (d). Axial FLAIR image (e) reveals that there is no lesion in the pons

T2-weighted and fluid attenuated inversion recovery (FLAIR) sequences revealed asymmetric extensive hyperintense areas in both frontoparietal and occipital lobes predominantly involving periventricular and subcortical hemispheric white matter. Post-contrast T1-weighted images showed no obvious contrast enhancement. There was mass effect with compression of the posterior horn of the lateral ventricle on the left side (Figure 1a–1e).

The patient became confused progressively on the following days. Lumbar puncture was performed and cerebrospinal fluid (CSF) was examined for infectious etiologies. JCV DNA was extracted from CSF using EZ1 automated extraction system (EZ1 virus mini kit v2.0, Qiagen, Hilden, Germany) and real-time PCR (RealStar JCV PCR Kit, Altona, Germany). Real-time PCR was positive for JCV DNA (viral load of 450,000 copies/mL). The patient was diagnosed with PML due to JC virus.

**Figure 2:**
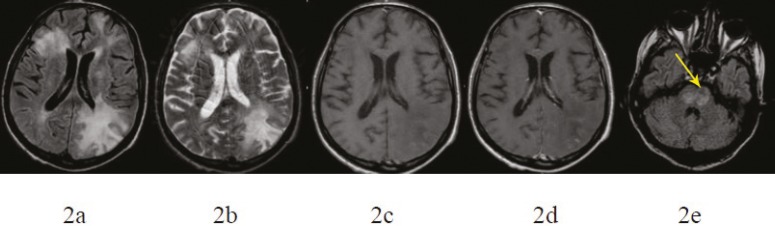
After immunosuppressive treatment withdrawal, axial FLAIR (a), T2-weighted (b), T1-weighted (c), and T1-weighted postcontrast (d) images show that the lesion extension and mass effect did not improve. Moreover, new lesions in the pons are appeared bilaterally, in keeping with progression (arrows) (e

After the diagnosis, the immunosuppressive regimen was discontinued and cytarabine was initiated at a dose of 2 mg/kg daily for five days. Unfortunately, the motor functions of the patient worsened on the following days. He was notable to do daily activities by himself without anybody. Three weeks later, a second MRI was obtained that showed the extension and character of the lesions and their mass effect had not been changed (Figure 2a–2e); also a new increased signal in the pons was seen bilaterally (Figure 2e). The patient’s condition got worse. The patient died at the fifth week of his last admission. 

## DISCUSSION

PML is a neurological demyelinating disorder caused by the JCV. The disease is a rare complication occurred after solid organ transplantation. The first reported cases of PML was diagnosed after renal transplantation [7]. So far eight cases of PML have been published in the literature that were diagnosed after liver transplantation [8-14]. The initial diagnosis of our case was cirrhosis secondary to chronic hepatitis B. Our case was the first patient who received three liver transplantations, two of which were complicated by by HAT. Then, the patient developed PML.

The incidence of CNS complications after liver transplantation is between 13% and 47%. Most of the neurological complications occur within the first two months. The etiology of these complications can be due to metabolic disorders, infections, or drug adverse effects [15]. White matter lesions of the CNS such as opportunistic infections, toxic effect of drugs, and metabolic disturbances can also be diagnosed in immunosuppressed individuals, with or without leukoencephalopathy. Differential diagnosis can be made taking into account the onset of these clinical conditions. PML can occur in liver transplant recipients in early and late post-transplantation periods [6]. PML usually manifests with sub-acute neurological deficits including altered mental status, motor deficits (hemiparesis or monoparesis), limb ataxia, gait ataxia, seizure and visual symptoms such as hemianopia and diplopia [16]. In our patient, quadriparesis and dysarthria were the first neurological findings. Then, his mental status disturbed and rapidly progressed to confusion.

Brain biopsy is the gold standard for the diagnosis of PML. Even if there is a neurological disease with white matter lesions on MRI and a positive PCR for the JCV in the CSF, the diagnosis of PML cannot be confirmed without brain biopsy [6]. The examination of CSF was positive for JCV DNA by PCR in our patient.

During the last decade, atypical radiological findings were reported for PML; those included ring contrast enhancement and mass effect [12]. Even the contrast enhancement of PML lesion is an atypical presentation. Some studies showed that these patients have better survival because of the development of an inflammatory response with breakdown of the blood-brain barrier [17]. MRI study of our patient showed no obvious contrast enhancement. However, it showed an atypical feature like mass effect that causes compression of the posterior horn of the lateral ventricle. Both of these radiological features were poor prognostic factors for our case.

JCV viral load is also another prognostic factor. A small study found that patients with a low viral load (50 to 100 copies/mL) in the CSF had a longer survival than patients with a higher viral load [18]. The load for our patient was very high—450,000 copies/mL.

So far, eight cases with PML due to JCV after liver transplantation have been reported in the literature. We report the ninth patient. There were seven female and two male patients. The mean age of the patients was 56 (range: 39–72) years. The causes of liver transplantation were cryptogenic cirrhosis, secondary biliary cirrhosis, and HCV- and HBV-related cirrhosis. It seems that there is no direct relation between the cause of liver failure and JCV. Six patients were diagnosed by PCR; the remaining patients were diagnosed by brain biopsy. The mean time from liver transplantation to development of neurological signs was 27 (range: 2–113) months according to data of seven patients. Therefore, it seems that PML due to JCV may occur as well in the early as in the late post-transplantation period. Two patients did not receive any treatment; the rest of the patients tapered immunosuppression. Four patients received cytarabine adenoside; one received peginterferon and cidofovir. Unfortunately, all the patients were lost after the diagnosis [8–14].

There is no effective treatment for PML. Several agents have been administered but they have no proven efficacy against JCV infection. Furthermore, the benefit from discontinuation of immunosuppression has not been proven, yet. However, it was observed that in few patients, cessation of or reduction in immunosuppressive drugs with antiviral therapy (cytarabine adenoside, cidofovir, mirtazapine and mefloquine) improved patients survival [6, 18, 19]. Cytarabine adenoside showed some effect in an open-label study on non-HIV PML patients, but it had no efficacy in a multi-center randomized open-label clinical trial on PML patients with HIV [6].We also stopped the immunosuppressive therapy for our case and administered cytarabine, but unfortunately these efforts were not enough to improve our patient’s clinical status.

Liver transplant recipients who receive long-term immunosuppression can develop PML due to reactivation of JCV. Certainly, all the liver recipients do not encounter the lytic infection of the CNS caused by JCV. We need to know whether re-transplantation or repetitive induction immunosuppression may cause this viral disease. If so, we should be aware of this complication after re-transplantation of liver to prevent or minimize poor clinical outcomes of PML.
